# Fertility preservation in breast cancer patients

**DOI:** 10.1007/s12282-013-0463-5

**Published:** 2013-03-24

**Authors:** Tadahiko Shien, Mikiya Nakatsuka, Hiroyoshi Doihara

**Affiliations:** 1 Breast Cancer Treatment and Reconstruction Center, Okayama University Hospital, 2-5-1 Shikata-cho, Kita-ku, Okayama, Okayama 700-8558 Japan; 2 Department of Obstetrics and Gynecology, Okayama University Hospital, Okayama, Japan

**Keywords:** Breast cancer, Fertility preservation, Systemic therapy

## Abstract

Systemic therapies have been shown to effectively improve prognosis in patients with breast cancer. However, such therapies also become increasingly harmful as their duration increases, and they have adverse effects on fertility and ovarian function. Fertility preservation (FP) is important in young adult cancer survivors who may wish to have children. In Japan, some cancer societies recommend that the potentially serious effects of systemic therapy on ovarian function should be explained to women with cancer, and they should be encouraged to undergo FP prior to commencing treatment. Still, as there are no official guidelines, many oncologists lack the required knowledge and mind-set to advise young breast cancer patients on fertility issues. Counseling of patients and their families might improve their understanding about the influence of such treatment on fertility and ensure effective FP. There are several FP methods that can be selected before beginning treatment, and these methods have both advantages and disadvantages. Young adults with breast cancer who want to bear children in the future must be provided with FP counseling, in addition to advice about breast cancer treatment and prognosis.

## Introduction

In 1986, the National Coalition for Cancer Survivorship (NCCS) defined living patients after cancer diagnosis as cancer survivors; survivors need numerous supportive measures because of the many social problems that they face, and these measures would greatly influence their families and friends [1]. These problems differ among individuals depending on their social background; therefore, provision of support cannot be based on any standard guidelines. Recently, the importance of cancer survivorship has received recognition owing to the needs of these patients. Oncologists should consider appropriate and more effective treatment strategies according to the individual’s needs and social background.

The number of breast cancer patients, including those younger than 40 years of age and premenopausal patients who want to bear children, is increasing every year in Japan. Young patients often have a good prognosis and live long recurrence-free lives following systemic therapy for breast cancer. However, the treatments employed exert many physical and psychological effects on young patients who hold important positions in their societies and families. In addition, the influence of the treatment on fertility and the fetus is of great importance to young cancer survivors who wish to start a family.

In contrast, an increasing number of women are marrying late in life, and advances in the field of perinatal and reproductive medicine have improved the safety of infertility treatment. Therefore, advanced reproductive techniques can be used in patients receiving systemic therapy who want to have children after breast cancer treatment. However, there are numerous important questions that need to be resolved by the collaborative efforts of oncologists and gynecologists before cancer survivors are encouraged to proceed with fertility preservation (FP).

## Influence of systemic therapy for breast cancer on fertility

Chemotherapy influences the ovarian function and induces amenorrhea. The frequency of chemotherapy-induced amenorrhea (CIA) differs according to the patient age and the chemotherapy regimen administered. The rate of irreversible CIA increases with the advancing age of the patient [2, 3]. The reported rates of CIA associated with the use of AC (anthracycline and cyclophosphamide) and CMF (cyclophosphamide, methotrexate, and 5-FU) are 30–50 % and 40–70 %, respectively. The risk of CIA increases with the addition of taxane in patients over 40 years of age [4, 5]. One very important issue that needs to be resolved is the lack of data on the rate of normal pregnancy or delivery after recovery from CIA in breast cancer survivors, and also on ovarian function after systemic therapy, which varies among patients of different races and environments. It is unclear if the data reported from European countries can be applied to Japanese patients.

Recently anti-müllerian hormone and inhibin B have been used as markers of ovarian function [6, 7]. Data need to be collected on Japanese patients and these (or other new) markers must be used to predict the reserve ovarian function after systemic therapy. In young patients receiving endocrine therapy after the primary operation who wish to bear children in the future, it would be very useful and important to clarify ovarian function after 5 years of treatment.

## Trend of FP in cancer patients

The practice of FP among cancer patients is closely related to the social background of the patients and entails several ethical problems. In Japan, embryo preservation among partners who are not married is not permitted. Age and prognosis are important considerations for FP. However, there are as yet no appropriate guidelines or consensus. Nevertheless, FP technologies and cancer therapy are improving every day, and the number of cancer survivors who want to have children is also increasing. Several Japanese hematologic and oncologic societies recommend the provision of accurate information concerning the influence of chemotherapy on fertility and FP to cancer patients and their families. In 2004, the Japan Society of Clinical Oncology, the Japan Society of Obstetrics and Gynecology, and the Japanese Urological Association suggested that oncologists and gynecologists should provide a thorough FP explanation to breast cancer patients before the start of systemic and radiation therapy. The Japan Society for Hematopoietic Cell Transplantation expressed concerns about giving their patients’ information (present technology and the limits of FP) to all members in 2005. This trend is similar to that observed in the USA. To assist healthcare professionals to inform their patients about FP, several national organizations, including the President’s Cancer Panel (2004), the American Society of Reproductive Medicine Ethics Committee (2005), and the American Society of Clinical Oncology (2006) [8], published FP guidelines [9]. The multi-institutional trial of oocyte cryopreservation for unmarried hematological cancer patients by A-PART (The International Association of Private Assisted Reproduction of Technology Clinics and Laboratories) started in Japan in 2007 (http://www.apartonline.info/japan/).

At the present time, Japanese cancer societies only recommend the “provision of appropriate information” to young patients, and there is no guiding principle or standard method for FP, which often involves special procedures that patients cannot receive at every hospital with public insurance in Japan. Moreover, the legal aspects of the ethical problems related to preservation of oocytes and sperm have not yet been discussed, and are only auto-regulated by the relevant societies in Japan.

## Current status of FP and counseling for breast cancer patients in Japan

The Japanese Breast Cancer Society and Clinical Practice Guidelines for Breast Cancer do not yet provide definitive recommendations for Japanese breast oncologists regarding FP for young breast cancer patients. In a previous report, significantly fewer patients expressed an interest in fertility when they had children or advanced disease, and patients with less aggressive treatment were recommended FP by oncologists [10]. However, from the results of our survey [11], the patient’s desire to give birth does not always depend on the number of children they have, age, and marital status, and we need to confirm their wishes individually before commencing treatment. We thought it necessary to establish a counseling system under which information can be supplied efficiently to breast cancer patients requiring FP, ensuring that patients who want to have children could undergo FP before receiving appropriate treatment for their breast cancer. Shimizu et al. [12] reported the results of a national survey on this problem, and many Japanese oncologists lacked the necessary knowledge and mind-set to advise young breast cancer patients on fertility issues. The barriers to discussing FP were risk of recurrence, lack of collaborating reproductive specialists, and time constraints in the clinic. Therefore, it is necessary to develop a comprehensive and interdisciplinary program for healthcare providers.

Many young cancer patients with breast cancer have little knowledge of fertility issues. This lack of knowledge is associated with increased decisional conflict; targeted and timely fertility information may reduce the patients’ concerns and increase informed choice [13]. However, this is very difficult for oncologists alone to achieve, and specialist teams in fertility counseling are needed.

## Details of FP

### Measures to reduce ovarian disorders caused by treatment

A gonadotropin-releasing hormone (GnRH) agonist is administered for a short period to suppress ovulation and to prevent reduction in ovarian function. Approximately 2 weeks after the start of the GnRH agonist treatment, the effect of the agonist in suppressing oocyte maturation begins to become apparent, making the oocytes less sensitive to chemotherapy. As it takes approximately 2–3 weeks for this effect to manifest, the GnRH agonist needs to be administered in a well-planned manner before the start of treatment for the malignant disease [14]. There are some ongoing studies to determine the exact nature of this process. The ZORO multicenter study reported in 2011 that there was no significant effect of GnRH on ovarian function after adjuvant chemotherapy [15]. There is no evidence for the validity of this technique as a tool for FP, and current guidelines do not recommend its use [16], even though it has been suggested to have the potential to reduce the likelihood of CIA [15].

### Storing frozen oocytes or ovarian tissue before the start of treatment

The validity of the measures taken for reducing ovarian disorders during treatment or post-treatment measures to deal with the loss of ovarian function is essentially limited, when their relationship to the malignant disease or its treatment is taken into account. The most promising approach is frozen storage of the patient’s oocytes or ovarian tissue collected before the start of systemic treatment. At present, research into this technology is underway with the aim of establishing a reliable system. Table 1 shows the details of each method proposed for storing oocytes and ovarian tissue. Although procedures for preserving of oocytes or the ovarian tissue itself are available, the collection of oocytes and/or ovarian tissue requires a preparatory period and involves physical stress (puncture and operation). Recent optimal success rates per cycle are reported to be in the 30–40 % range or higher per embryo transfer. According to the American Society of Reproductive Medicine (ASRM), on the basis of published peer-reviewed medical literature, there is an approximate overall 2 % live-birth rate per oocyte thawed for cryopreservation by using the older slow-freeze methods. There is an approximate overall 4 % live-birth rate per oocyte thawed for cryopreservation by using vitrification. However, pregnancy rates as high as 8 % per frozen egg (on average 12.4 eggs needed per pregnancy) have been achieved at centers using some of the newer freeze methods such as LANDA (http://donor-egg-program-utah.fertilitydr.com/egg-freezing-overview.html).

**Table 1:** Fertility preservation options for breast cancer patients

	Option		
	Oocyte freezing	Embryo freezing	Ovarian tissue freezing
Method	Ovarian puncture	Ovarian puncture	Laparoscopic surgery
Depending on menstruation	Yes	Yes	No
Delay of cancer treatment	3–4 weeks	3–4 weeks	Several days
Follicle stimulation	Necessary	Necessary	Unnecessary
Number of preservation eggs	1–10	1–10	Many
Partner sperm	Unnecessary	Necessary	Unnecessary
IVF-ET	Necessary	Necessary	Unnecessary
Implantation of cancer cell	No	No	Yes
Success rate	10–30 %	10–40 %	Unestimated

*IVF-ET* in vitro fertilization and embryo transfer

Oocyte preservation can be accomplished by either oocyte cryopreservation or embryo cryopreservation. In general, the oocyte is more susceptible to damage caused by cryopreservation, and fertility is lower with oocyte cryopreservation than with embryo cryopreservation. For married women, storage of embryos after in vitro fertilization is possible. For unmarried women, however, unfertilized oocytes must be selected because of the unavailability of sperm from a fixed partner. In recent years, following active research and development on the methods for cryopreservation and storing of unfertilized oocytes, the number of facilities providing such services has increased worldwide [17, 18]. In Japan, the first such attempt was reported in 2001 (for a married couple who successfully bore a child using this technique).

These techniques involve two major problems when applied to breast cancer patients. The first problem pertains to the use of follicle stimulation to facilitate oocyte collection and can cause elevation of blood estrogen levels, even if only temporarily. In healthy women, this manipulation has not been considered to have the potential to increase the risk of breast cancer, gynecologic cancers, etc. [19–21], and pregnancy after estrogen receptor (ER)-positive breast cancer does not seem to increase the risk of recurrence [22]; however, the influence of follicle stimulation on the prognosis of ER-positive breast cancer patients is unclear [23, 24]. The special follicle stimulation method for breast cancer or its treatment is being examined, but as it is still at the stage of basic research, no clinical evidence has been obtained yet [25]. At present, it is recommended that this procedure should be done after completion of at least local surgery.

The second problem pertains to the timing of the FP, which should be planned without affecting the cancer treatment plan. To collect oocytes, follicle stimulation takes place according to a schedule that matches the menstrual cycle of each patient. Therefore, in some cases, approximately a month may be needed to collect oocytes depending on the menstrual cycle. Thus, adjustment of the timing of treatment and FP may be difficult in cases where the treatment is urgently needed. It is, therefore, difficult to set aside a period for FP before preoperative chemotherapy in cases of local advanced breast cancer that require prompt treatment. As the breast cancer treatment strategy is highly influenced by the FP procedure, oncologists need to be conscious of the patient’s desire to bear children before the start of treatment.

Ovarian tissue cryopreservation has begun to attract attention as a new approach [26]. Its main advantages are that it does not involve follicle stimulation and that it enables preservation by a quick collection of a part of the ovary by an obstetrician/gynecologist at a facility where laparoscopic surgery is possible. This approach obviates the need for adjustment of treatment time and scheduling according to the patient’s menstrual cycle. If the stored ovarian tissue is implanted subcutaneously into the forearm, abdominal wall, etc., additional procedures such as in vitro fertilization and embryo transfer (IVF-ET) would be needed. However, if it is implanted laparoscopically onto a site near the oviduct, spontaneous pregnancy can be expected. However, according to reports, care needs to be taken for the possible presence of tumor cells in the ovarian tissue [27]. In Japan, clinical research on the storage of ovarian tissue has been ongoing at Okayama University since 2005. Although there are ethical and technical aspects that must be resolved before it can be widely adopted in Japan, this approach is promising in view of the following features: (1) If the thawed ovarian tissue survives after implantation, pregnancy and secretion of female hormones can be expected; (2) follicle stimulation is unnecessary; and (3) a larger number of stored oocytes can be obtained, possibly elevating the fertility rate.

## Conclusion

The demand for provision of FP to young adult breast cancer patients receiving systemic therapy is increasing, and FP techniques are steadily improving. It is very difficult to decide the eligibility of breast cancer patients for FP because of differences in the individual’s situation and environment. Therefore, an active counseling system tailored to each patient must be devised by the respective attending specialists. There are many unresolved technical, ethical, and social problems in this field. We must provide appropriate explanations of these issues to young adult breast cancer patients who want to have children and make appropriate treatment plans to preserve their fertility, in addition to explaining the cancer treatment and outcomes to these patients.
